# Association of tumor associated collagen signature with lymph node metastasis in pancreatic ductal adenocarcinoma

**DOI:** 10.1002/btm2.70087

**Published:** 2025-10-30

**Authors:** Gangqin Xi, Linying Chen, Xiwen Chen, Yuhang Huang, Junyang Luo, Jiajia He, Xiaolu Li, Jianxin Chen, Guozhong Liu, Lianhuang Li, Shuangmu Zhuo

**Affiliations:** ^1^ School of Science Jimei University Xiamen China; ^2^ Department of Pathology The First Affiliated Hospital of Fujian Medical University Fuzhou China; ^3^ Key Laboratory of OptoElectronic Science and Technology for Medicine of Ministry of Education, Fujian Provincial Key Laboratory of Photonics Technology Fujian Normal University Fuzhou China; ^4^ Department of Clinical Medicine Xiamen Medical College Xiamen China; ^5^ Department of Hepatopancreatobiliary Surgery the First Affiliated Hospital of Fujian Medical University Fuzhou China

**Keywords:** lymph node metastasis, multiphoton microscopy, pancreatic ductal adenocarcinoma (PDAC), predict, tumor associated collagen signature (TACS)

## Abstract

Pancreatic ductal adenocarcinoma (PDAC) is a highly aggressive and fatal cancer with significant metastatic potential. Lymph node status is crucial for determining treatment options and predicting prognosis in pancreatic cancer patients. Current methods for estimating lymph node metastasis in PDAC are inadequate. This study developed and validated a novel nomogram that integrates macroscopic and microscopic tumor‐associated collagen signatures (ma‐TACS and mi‐TACS) within the tumor microenvironment to assess the risk of lymph node metastasis in PDAC patients. This retrospective study included 150 PDAC patients, with 92 in the training cohort and 58 in the validation cohort. Ma‐TACS and mi‐TACS were obtained by multiphoton microscopy. Mi‐TACS, which includes both morphological and textural features, were extracted from segmented regions of interest using Matlab 2022a. Ma‐TACS and mi‐TACS scores were calculated using ridge regression and LASSO regression analysis. Ma‐TACS and mi‐TACS scores are significantly related to lymph node metastasis in both univariate and multivariate logistic regression analyses (ma‐TACS score, odds ratio, 2.304; 95% CI, 1.412–3.761; *p* = 0.001; 2.934, 1.409–6.108, *p* = 0.004; mi‐TACS score, odds ratio, 3.325; 95% CI, 2.296–4.814; *p* < 0.001; 3.861, 2.488–5.993, *p* < 0.001). The nomogram model, integrating the ma‐TACS and mi‐TACS scores, successfully stratified patients into lymph node negative and positive groups, achieving areas under the curve of 0.918 in the training cohort and 0.831 in the validation cohort. The results indicate that the tumor‐associated collagen signatures independently predict lymph node metastasis in PDAC. Additionally, the prediction model based on TACS may be valuable in guiding treatment decisions for PDAC patients.

**Translational Impact Statement**

This study shows that label‐free multiphoton microscopy can reveal tumor‐associated collagen signatures (ma‐TACS and mi‐TACS) within the pancreatic ductal adenocarcinoma microenvironment, which are strongly associated with lymph node metastasis. The nomogram integrating these features demonstrated strong predictive performance and holds potential clinical value for guiding treatment strategies and improving prognosis prediction in pancreatic cancer.

## 1 INTRODUCTION

Pancreatic cancer is highly fatal, with mortality rates nearly matching the incidence rates.^1^, ^2^ Pancreatic ductal adenocarcinoma (PDAC) is the most common subtype, constituting 85%–90% of all pancreatic cancer patients.^3^ Despite advancements in medical technology, the 5‐year overall survival rate for PDAC remains only 6%, due to poor diagnosis and prognosis.^4^ Surgical resection is the only treatment with curative potential, leading to significantly longer survival compared to other treatments. Over 70% of resected ductal adenocarcinomas exhibit lymph node (LN) metastases, even if the primary tumor is smaller than 2 cm.^5^ LN status is a significant predictor of survival in pancreatic cancer resection, determining the surgical procedure and patient outcomes.^6^ LN dissection is usually unavoidable for most patients undergoing pancreatic cancer surgery to achieve radical resection. Some patients without LN metastasis still undergo LN dissection and its associated risks. Therefore, accurately assessing LN status in pancreatic cancer is essential for tailored surgical procedures.

Several imaging techniques, including computed tomography (CT), magnetic resonance imaging (MRI), and positron emission tomography (PET), can aid in preoperatively assessing LN status in pancreatic cancer.^7^, ^8^ These imaging methods evaluate LN status based on morphology, such as variations in size, shape, and intensity, often leading to high false‐positive rates. Conversely, normal‐sized LNs can contain micrometastases, causing high false‐negative rates.^9^ These approaches fail to offer effective clinical guidance, and the imaging features are inadequate as predictive factors. Pancreatic cancer with a higher tumor grade, larger size, and lymphovascular invasion is considered high risk for LN metastasis, suggesting the need for radical surgery, though consensus is lacking.^10^ Various models have been developed to estimate the likelihood of LN metastasis in pancreatic cancer.^11^, ^12^ However, these models primarily focus on clinicopathologic variables and did not investigate the association between the tumor microenvironment and LN metastasis.

The extracellular matrix (ECM) forms the scaffold of the tumor microenvironment, playing a crucial role in regulating cancer behavior. Collagen fibers, glycoproteins, and proteoglycans are its main structural components.^13^, ^14^ Collagen fibers, a key ECM component, can influence cell movement by either acting as barriers to invasion, or depending on their alignment, as pathways for rapid tumor cell migration.^15^, ^16^ PDAC is characterized by fibrotic tissue with an abundant ECM surrounding the tumor. This extensive fibrotic stroma can constitute up to as much as 90% of the overall tumor volume.^17^ Fibrillar collagens constitute the majority of PDAC stroma and are implicated in tumor cell migration, proliferation, and invasion.^18^ However, the specific role of collagen fibers in LN metastasis in PDAC remains uncertain.

The development of nonlinear microscopy, such as multiphoton microscopy (MPM), has allowed for the visualization of collagen fiber changes at cellular resolution.^19^, ^20^ MPM offers detailed tissue architecture insights in unprocessed specimens by combining two‐photon excitation fluorescence (TPEF) with second harmonic generation (SHG). SHG imaging provides a direct, label‐free method for visualizing collagen structures. Collagen fibers have a distinctive non‐centrosymmetric structure, allowing them to function as frequency doublers when exposed to multiphoton laser light. Using coherent, nonabsorptive light interaction, individual collagen fibers can be imaged at high resolution without external staining.^21^, ^22^ Several groups have shown that alterations in collagen fiber structure and organization during tumorigenesis have significant biological implications. These changes are associated with clinical outcomes across various types of solid tumors.^23^, ^24^, ^25^ Specifically, several collagen organization patterns, known as tumor‐associated collagen signatures (TACS), have been recognized in breast tumors and have significant implications for disease progression.^24^, ^26^, ^27^ In our prior study, we extended the original TACS1–3 by identifying additional patterns, TACS4–8, on a larger scale, specifically at the invasion front of primary breast tumors. Notably, we established a strong correlation between various TACSs and breast cancer prognosis.^26^ Furthermore, these structural patterns are not exclusive to breast cancer. Recent studies indicate that such collagen structures are also prevalent in PDAC, another highly metastatic desmoplastic disease.^28^ Despite recent work indicating the presence of TACS structures in PDAC, the relationship between TACS and PDAC progression, particularly in relation to LN metastasis, remains largely unexplored. Building on our previous study, we investigated the relationship between macroscopic TACS and LN metastasis. Recently, extracting detailed quantitative features from SHG images has become a rapidly advancing field, helping uncover links between microscopic collagen features and various pathological conditions.^29^, ^30^ Thus, to better understand the relationship between subtle changes in TACS and LN metastasis in PDAC, we extracted microscopic TACS features (mi‐TACS) and combined them with ma‐TACS. This provided a more comprehensive understanding of how collagen signatures relate to LN metastasis. Finally, we developed and validated a nomogram that integrates both ma‐TACS and mi‐TACS from MPM imaging to provide personalized predictions of LN metastasis in PDAC patients.

## 2 RESULTS

### 2.1 Patients' characteristics

Based on the selection criteria, 150 patients were randomly assigned into a training cohort (*n* = 92) and a validation cohort (*n* = 58). Although the randomization process was not stratified, it ensured a reasonably balanced distribution. No significant differences were observed between the two cohorts in terms of PDAC LN status or baseline clinicopathological characteristics, including age, gender, tumor size, differentiation grade, lymphovascular invasion, perineural invasion, and tumor location (*p* > 0.05 Table S1). Table 1 summarized the clinical characteristics and collagen scores distribution of the LN0 and LN1 groups. Among the LN0 and LN1 groups in the training cohort, the ma‐TACS score and mi‐TACS score were significantly different (*p* < 0.05). In the validation, the ma‐TACS score was close to being significantly different between LN0 and LN1 groups (*p* = 0.080).

**TABLE 1:** Characteristics of patients with PDAC in the LN metastasis negative (LN0) and LN metastasis positive (LN1) groups.

Characteristic	Training cohort			Validation cohort		
	LN0 (*n* = 47)	LN1 (*n* = 45)	*p*‐Value	LN0 (*n* = 26)	LN1 (*n* = 32)	*p*‐Value
Age			0.287			0.339
≤60	23 (48.9%)	27 (60.0%)		13 (50%)	12 (37.5%)	
>60	24 (51.1%)	18 (40.0%)		13 (50%)	20 (62.5%)	
Gender			0.555			0.479
Male	14 (29.8%)	16 (35.6%)		9 (34.6%)	14 (43.8%)	
Female	33 (70.2%)	29 (64.4%)		17 (65.4%)	18 (56.3%)	
T category			0.560			0.197
T1	5 (10.6%)	8 (17.8%)		3 (11.5%)	3 (9.3%)	
T2	27 (57.5%)	22 (48.9%)		12 (46.2%)	22 (68.8%)	
T3	15 (31.9%)	15 (33.3%)		11 (42.3%)	7 (21.9%)	
Differentiation grade			0.661			0.115
G1	7 (14.9%)	7 (15.6%)		8 (30.8%)	5 (15.6%)	
G2	18 (38.3%)	21 (46.7%)		11 (42.3%)	10 (31.3%)	
G3	22 (46.8%)	17 (37.7%)		7 (26.9%)	17 (53.1%)	
Lymphovascular invasion			0.379			0.980
Negative	39 (83.0%)	34 (75.6%)		22 (84.6%)	27 (84.4%)	
Positive	8 (17.0%)	11 (24.4%)		4 (15.4%)	5 (15.6%)	
Perineural invasion			0.907			0.472
Negative	11 (23.4%)	11 (24.4%)		6 (23.1%)	5 (15.6%)	
Positive	36 (76.6%)	34 (75.6%)		20 (76.9%)	27 (84.4%)	
Tumor location						0.221
Head 1	31 (66.0%)	33 (73.3%)	0.710	17 (65.4%)	27 (84.4%)	
Body/tail 2	14 (29.8%)	10 (22.2%)		8 (30.8%)	4 (12.5%)	
Other 3	2 (4.2%)	2 (4.5%)		1 (3.8%)	1 (3.1%)	
Ma‐TACS score, median (IQR)	−0.377 (−0.614 to 0.148)	0.130 (−0.289 to 0.846)	0.002	−0.639 (−1.000 to 0.004)	−0.086 (−0.757 to 0.437)	0.080
Mi‐TACS score, median (IQR)	−1.262 (−1.756 to 0.406)	1.070 (0.386 to 1.383)	<0.001	−1.584 (−2.279 to −0.425)	0.750 (0.124 to 1.257)	<0.001

Abbreviation: IQR, interquartile range.

### 2.2 Collagen feature extraction, signature score construction

**FIGURE 1:**
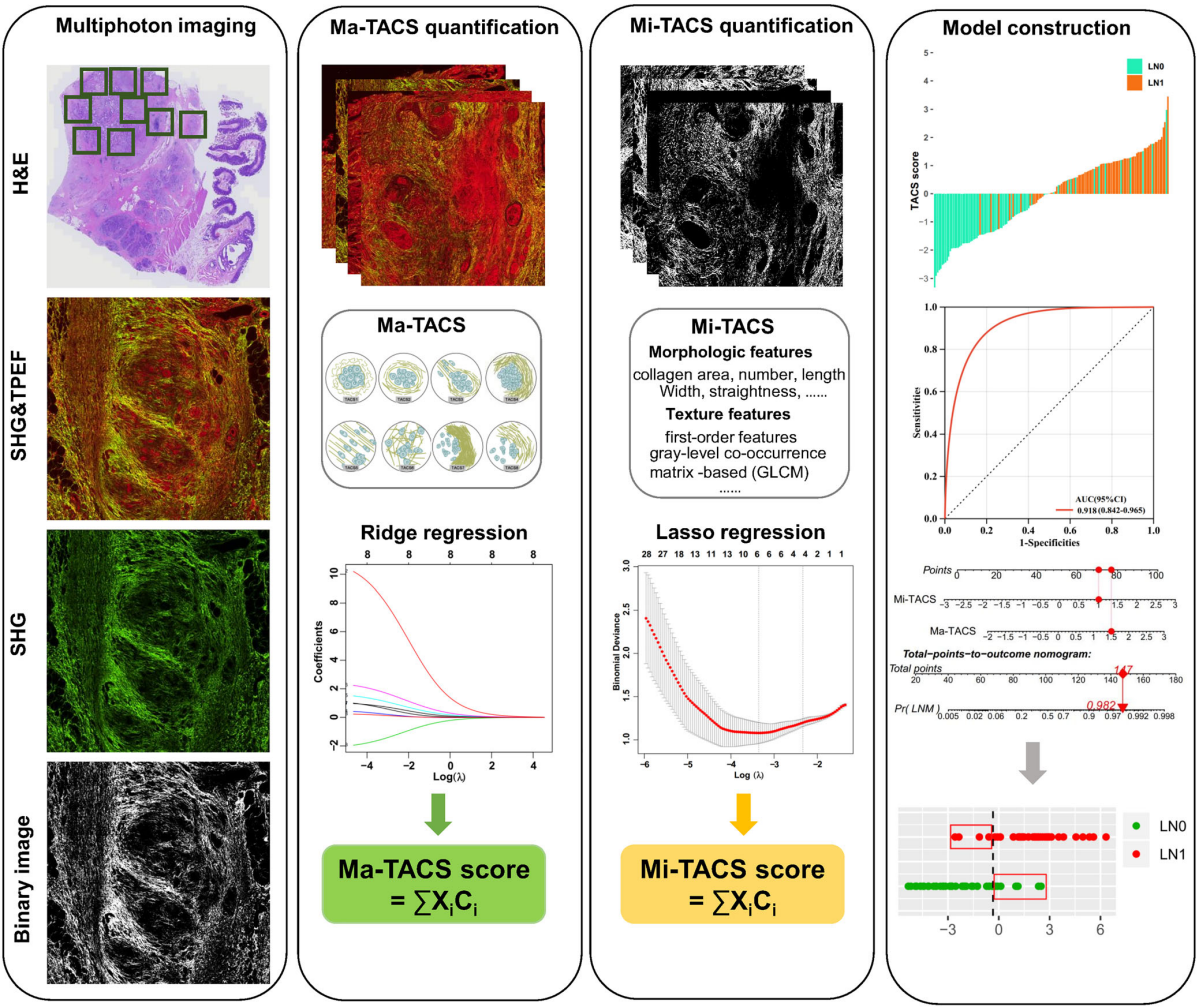
Study flowchart. The images in the left panel, from top to bottom, show the H&E image whole section with the region of interest (ROI) highlighted, the MPM image of a specific ROI (including TPEF and SHG), the corresponding SHG image, the binary images converted from the SHG image. Ridge and Lasso regression were then used to quantify the collagen features, and a model incorporating these collagen scores was then developed and validated.

Using MPM images, we identified eight major TACSs, similar to the way histopathological subtypes are identified from H&E images. As for TACS1–8, they were primarily based on macroscopic morphological changes of collagen in the tumor microenvironment. As illustrated in Figure 1, the ma‐TACS score, which is related to LN metastasis, represents a combination of the eight identified TACSs. In Data S1, we provide the formula for the ma‐TACS score. We segmented the SHG image and extracted 142 mi‐TACS features. A LASSO logistic regression analysis was applied to the training cohort to identify six LN metastasis‐related features (Figure S1). The formula for calculating the mi‐TACS score is provided in Data S1.

### 2.3 Predictive performance of the ma‐TACS score and mi‐TACS score

**FIGURE 2:**
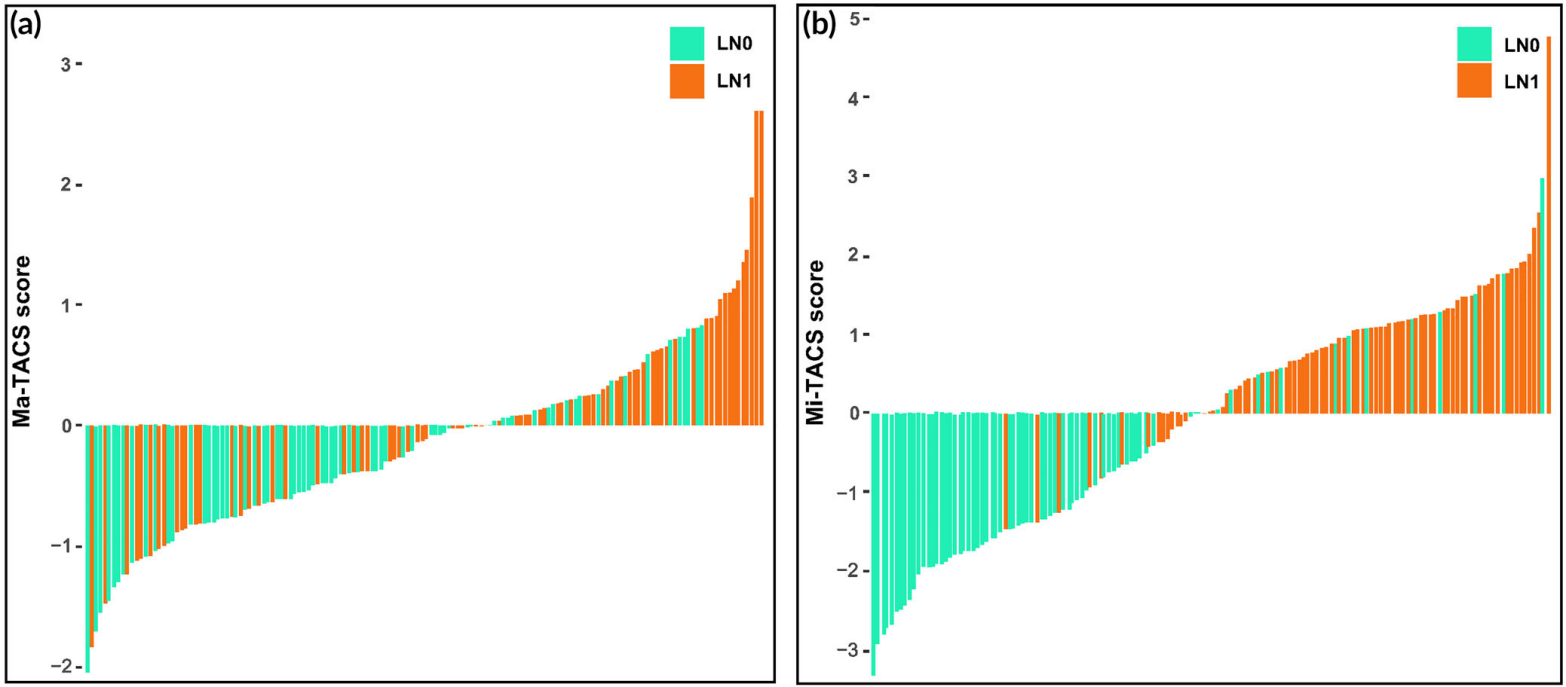
(a) Ma‐TACS and (b) mi TACS scores of each patient. LN0 or LN1 patients are labeled with different colors, where LN0 indicates the absence of lymph node metastasis, and LN1 indicates the presence of lymph node metastasis.

Univariable and multivariable logistic regression analyses revealed a significant correlation between ma‐TACS score, mi‐TACS score, and LN metastasis (Table S2). A histogram of ma‐TACS and mi‐TACS scores in LN0 and LN1 was shown in Figure 2, with high scores consistently linked with LN1 and low scores with LN0. High scores were mostly LN1, while low scores were LN0. The scores of LN1 (orange box) were clearly differentiated from those of LN0 (green box). There were statistically significant differences in the ma‐TACS and mi‐TACS scores (interquartile range [IQR]) between the LN1 and LN0 groups (ma‐TACS score: −0.406 [−0.770 to 0.060] vs. −0.079 [−0.545 to 0.570], *p* < 0.05; mi‐TACS score: −1.341 [−1.802 to −0.462] vs. 0.952 [0.283 to 1.314], *p* < 0.05) (Figure S2).

The model's performance was mainly assessed through receiver operating characteristic (ROC) analysis conducted on both the training and validation cohorts. The ma‐TACS score achieved an area under the curve (AUC) of 0.687 (95% CI, 0.587–0.779) in the training cohort and 0.636 (95% CI, 0.499–0.758) in the validation cohort for predicting LN metastasis (Figure S3C, D). The mi‐TACS score showed a strong predictive ability with an AUC of 0.884 (95% CI, 0.800–0.941) in the training cohort and 0.831 (95% CI, 0.709–0.916) in the validation cohort (Figure S4E, F). The ma‐ and mi‐TACS model outperformed the clinical model (0.642; 95% CI, 0.535–0.739; 0.572; 95% CI, 0.435–0.701) (Figure S3A, B). Although a comprehensive clinical model was developed using multivariate logistic regression that incorporated key clinicopathological factors (age, gender, T category, differentiation grade, lymphovascular invasion, perineural invasion, and tumor location) and generated clinical scores through weighted coefficients, it failed to achieve superior predictive performance compared with the established ma‐TACS and mi‐TACS scoring systems.

**FIGURE 3:**
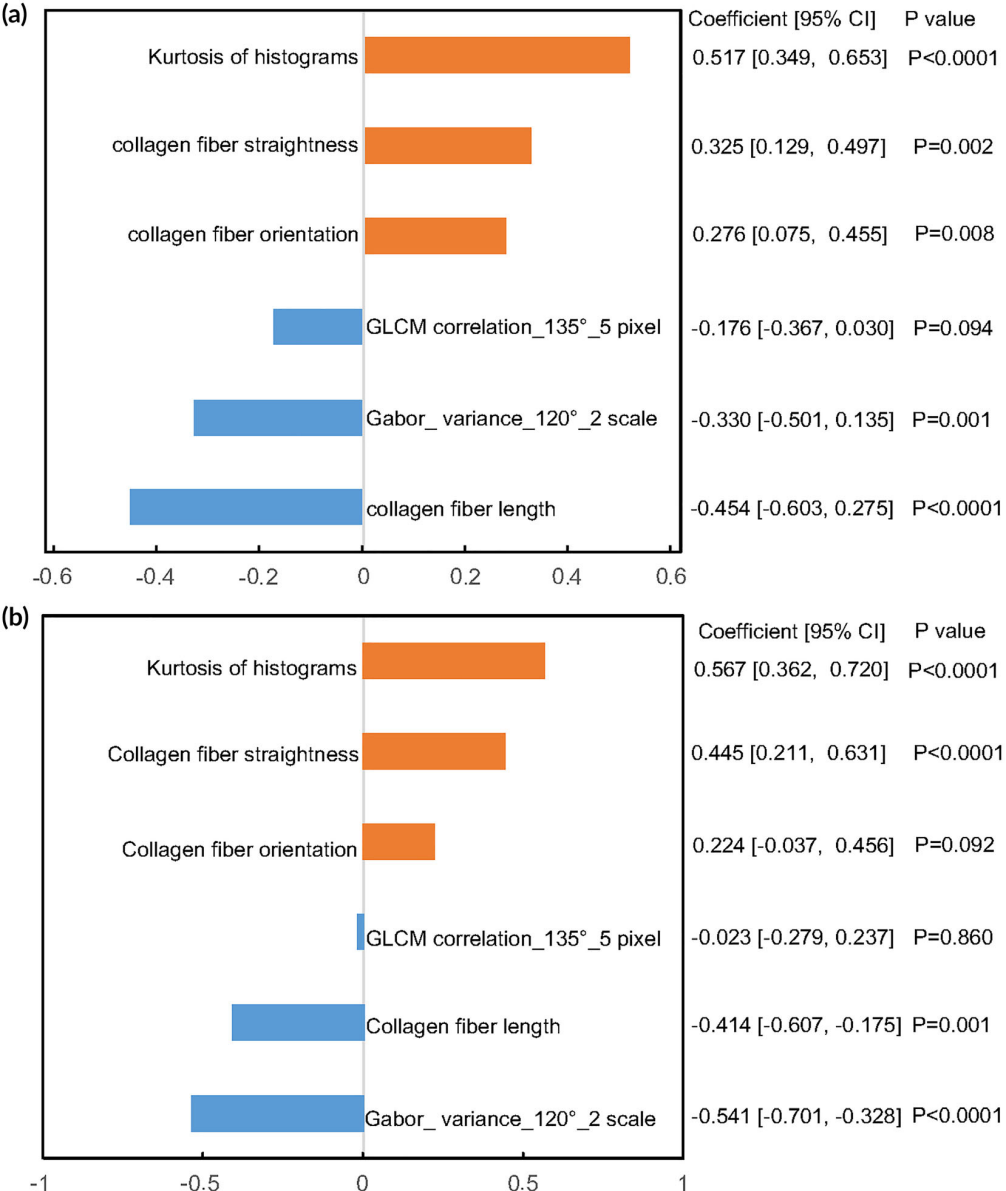
Correlation analysis between six selected collagen features by the LASSO regression and lymph node metastasis in the training (a) and validation cohort (b).

In contrast, mi‐TACS score exhibited a best prediction effect. The six collagen features selected by the LASSO regression to construct the mi‐TACS score are strongly correlated with LN metastasis (Figure 3). From the perspective of morphological features, the orientation angle and straightness of collagen fibers are also positively correlated with LN metastasis, while the length of collagen fibers is negatively correlated with LN metastasis.

### 2.4 Performance comparison of different prediction models

**FIGURE 4:**
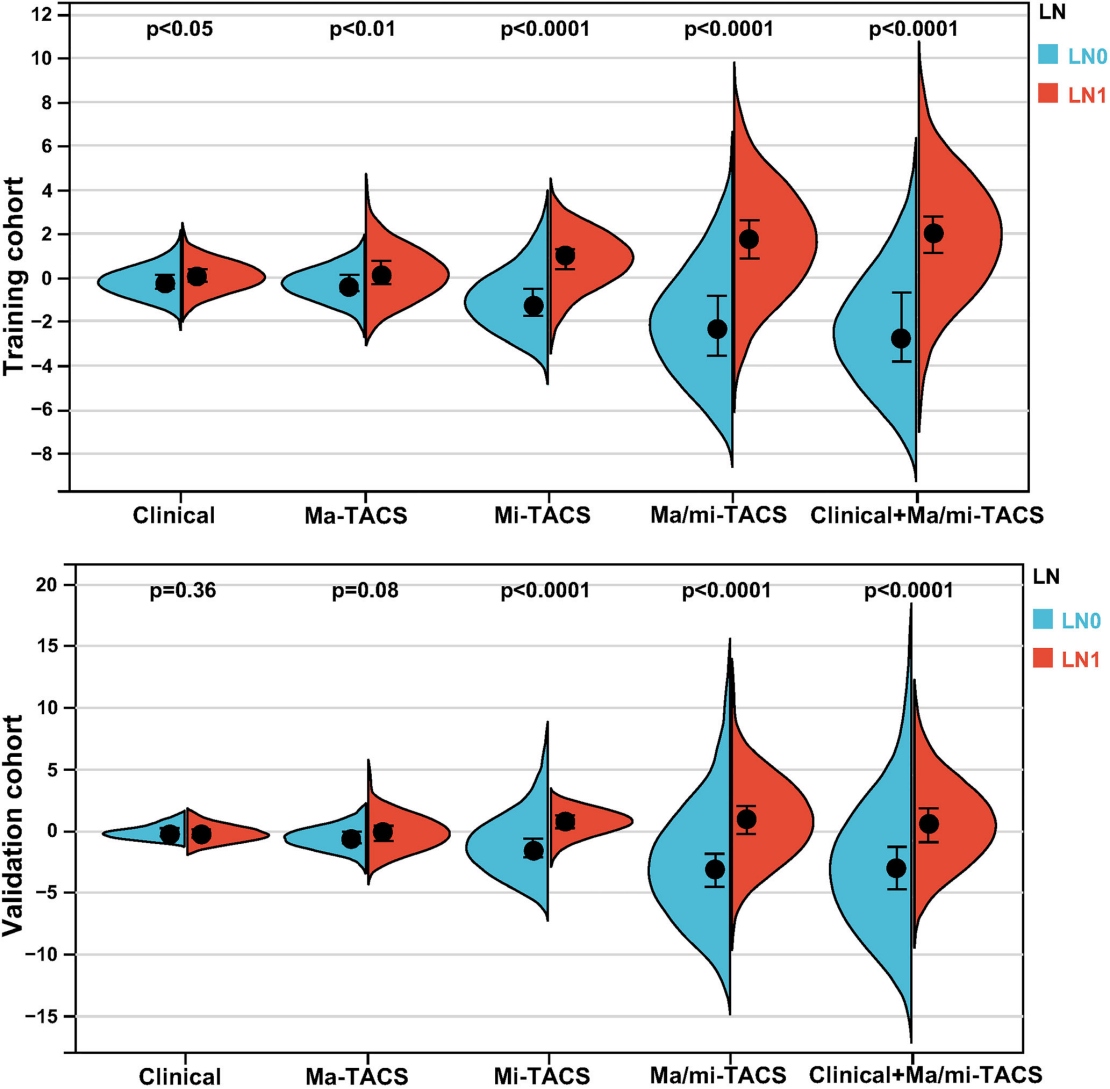
The violin plot displays the score differences between the LN0 and LN1 groups across the five models in both the training and validation cohorts.

To further assess the predictive performance of various models, we established five models to predict LN metastasis of PDAC (clinical, ma‐TACS, mi‐TACS, ma/mi‐TACS, clinical + ma/mi‐TACS). The predictive model established by clinicopathological factors (clinical model) exhibited the worst performance of the five models. There was a significant difference in the clinical model between LN0 and LN1 groups in the training cohort (*p* < 0.05), but no significant difference was observed in the validation cohort (*p* = 0.360). The ma‐TACS model was close to being significantly different (*p* = 0.080, *p* < 0.1) between LN0 and LN1 groups in the validation. In both training and validation cohorts, the mi‐TACS model demonstrated significant differences between the two groups (*p* < 0.001). The TACS combined (ma‐/mi‐TACS) model and the full model (clinical + ma‐/mi‐TACS) also showed significant differences in both cohorts (Figure 4).

When the ma‐TACS and mi‐TACS models are combined, the AUC increases to 0.918 (95% CI, 0.842–0.965) in the training cohort; however, there was no increase compared to the mi‐TACS model alone in the validation cohort (Figure S4G, H). This might be due to the mediocre performance of the ma‐TACS model. The combination of three single models instead reduced the predictive performance in the validation. Indeed, this is related to the poor predictive performance of the clinical model (Figure S5I, J). The corresponding sensitivity and specificity of the five models are presented in Table S3. Among the single models, mi‐TACS exhibited the best performance, demonstrating the highest sensitivity and specificity. Combined models, such as the full model, showed improved specificity but decreased sensitivity, especially in the validation cohort.

**FIGURE 5:**
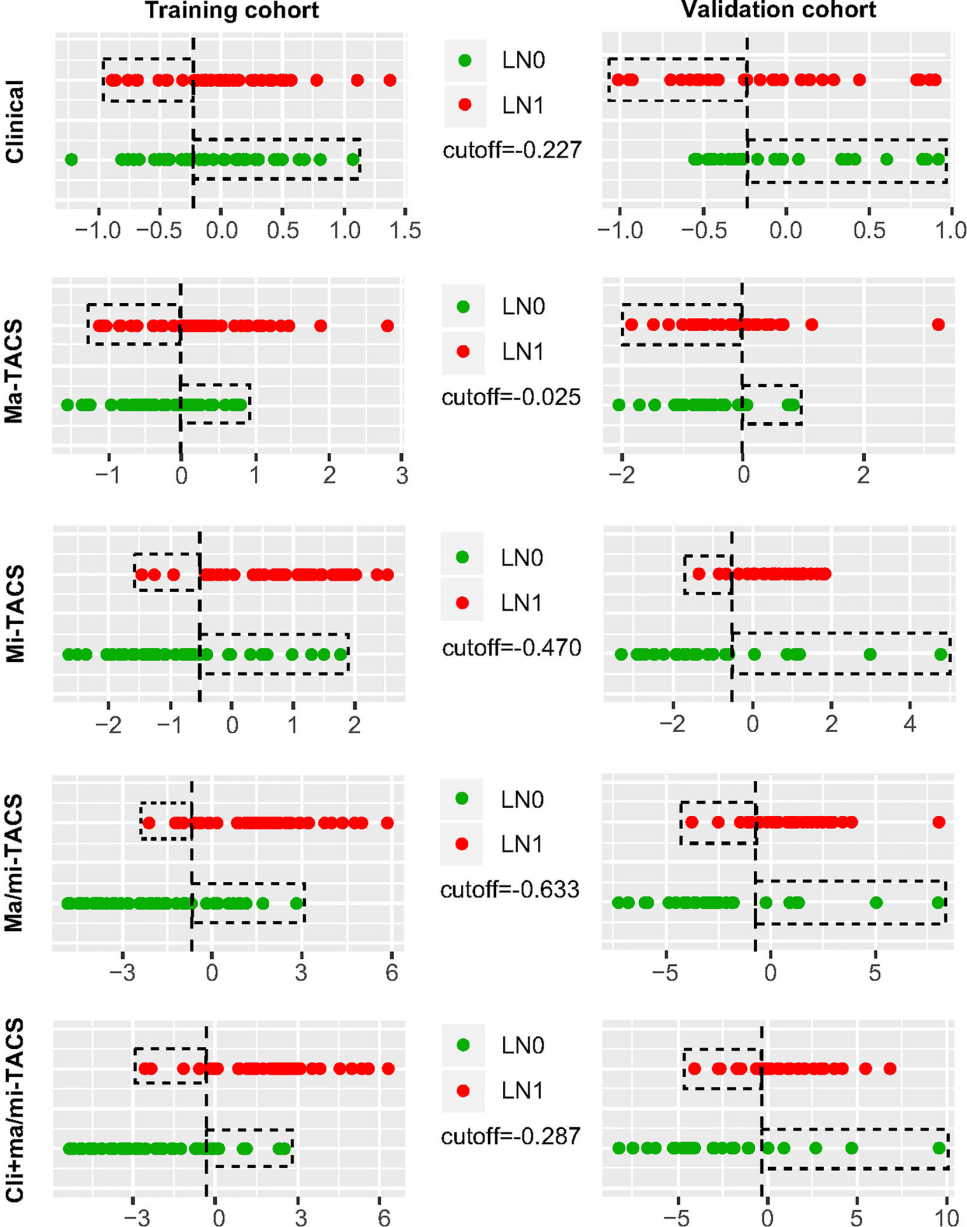
The prediction results of five models for lymph node metastasis in the training and validation cohorts.

The patients were stratified into LN0 and LN1 groups using thresholds derived from the training cohort based on the maximal Youden Index (sensitivity + specificity − 1), which identifies the optimal balance between sensitivity and specificity for each model. These thresholds were then applied to the validation cohort. The classification effect of the five models based on their respective thresholds can be seen intuitively from Figure 5. Left of the dashed line are the predicted LN0 patients, and right are the predicted LN1 patients. Green dots represent actual LN0 patients, while red dots indicate actual LN1 patients. The markers within the black dashed box highlight patients with incorrect LN classification. As can be seen in Figure 5, the clinical model performs worse in terms of specificity, that is, it is more likely to misclassify patients without LN metastasis as having metastasis (evidenced by the numerous green dots in the red box on the right), potentially leading to overtreatment. The ma‐TACS model has poor sensitivity, leading to more true LN metastasis patients being misclassified as non‐metastatic (as indicated by the numerous red dots in the red box on the left), potentially resulting in undertreatment. The mi‐TACS model outperforms the previous two single models, especially in terms of sensitivity, with only a few true LN metastasis patients being misclassified as non‐metastatic. The ma/mi‐TACS combined model and the full model both show further improvement.

### 2.5 Combined nomogram construction

**FIGURE 6:**
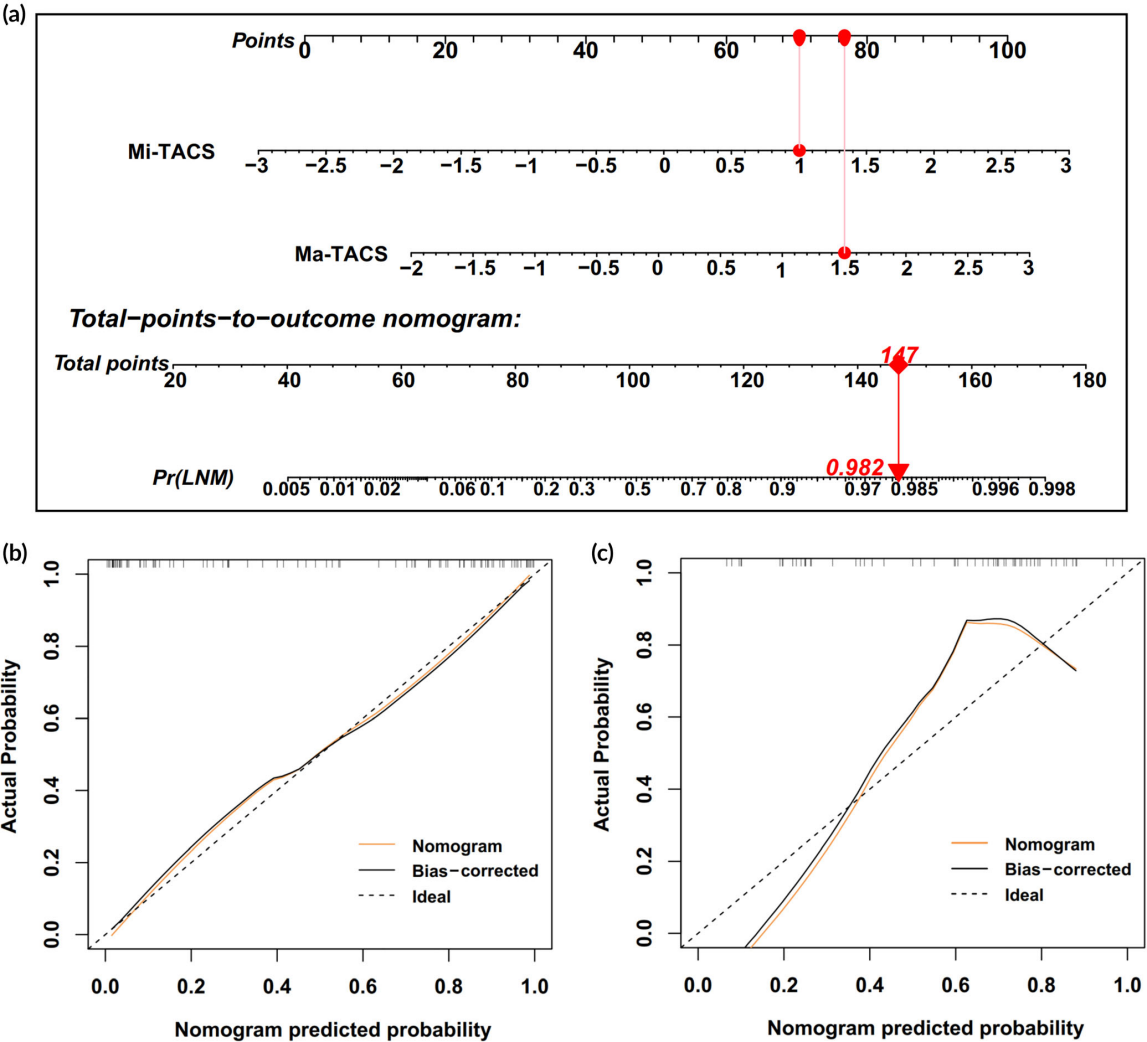
(a) Nomogram integrating ma‐TACS and mi‐TACS scores for prediction. (b, c) Calibration curves of the nomogram for both training (b) and validation (c) cohorts.

Furthermore, a clinically applicable nomogram integrating the ma‐TACS score and mi‐TACS score was developed to predict the risk of LN metastasis (Figure 6a). Each ma‐ or mi‐TACS score was assigned a corresponding point on the point scale. For example, if a patient had a mi‐TACS score of 1 and a ma‐TACS score of 1.5, they would receive a total of 147 points, indicating a 98.2% likelihood of LN metastasis. Additionally, the calibration charts of the training and validation cohorts indicate good consistency (C‐index: 0.918 and 0.831, respectively). The calibration curves for the nomogram can be seen in Figure 6b and c. To further clarify the predictive performance of the ma + mi‐TACS score across various subgroups, we performed several subgroup analyses based on clinical factors. We found that the predictive performance remained consistently strong across all subgroups (Table S4). The clinical decision utility of the predictive models was demonstrated using decision curve analysis (DCA). Figure S6A, B illustrated the clinical utility of the respective models in both cohorts based on the area under the decision curves. In both the training cohort and validation cohort, the nomogram model (ma‐/mi‐TACS, green) showed more area than the other models (ma‐TACS score, red; mi‐TACS score, orange; clinical, black), outperforming the “treat all” (gray) and “treat none” (blue) strategies. The nomogram model demonstrated the highest net benefit compared to the single models (clinical, ma‐TACS, mi‐TACS), indicating it is a reliable clinical tool for predicting LN metastasis in PDAC patients who had surgical resections.

## 3 DISCUSSION

The study showed a link between macroscopic and microscopic tumor‐associated collagen signatures (ma/mi‐TACS) and LN metastases in PDAC patients. The ROC analysis revealed that the nomogram model had excellent predictive capability in both the training and validation cohorts. The ma/mi‐TACS model (0.918, 0.831) and the single mi‐TACS model (0.884, 0.831) had significantly higher AUCs than the clinical model (0.642, 0.572). Univariate and multivariate logistic regression analyses identified both ma‐TACS and mi‐TACS scores as independent predictors of LN metastasis. Additionally, no clinicopathological factors were independently associated with LN metastasis in the logistic regression analysis. These results highlight the challenge of identifying PDAC patients at high risk for LN metastasis solely through clinical data assessment. The mi‐TACS model alone showed strong predictive performance. During LASSO optimization for mi‐TACS selection, the six selected features showed strong correlations with LN metastasis (Figure 3). Microscopic collagen features in pancreatic tumors better reflect tumor progression and invasion. Among them, the kurtosis of the first‐order histogram, collagen fiber straightness, and collagen fiber orientation were positively correlated with LN metastases, while collagen fiber length was negatively correlated. Geovanni et al. showed that collagen fibers in tumor tissue are more aligned and shorter compared to normal tissues.^31^ Previous studies indicated that collagen fiber orientation and straightness are prognostic markers in patients with invasive breast cancer.^24^, ^27^ Additionally, studies on pancreatic tumors found that highly aligned stromal collagen is a poor prognostic factor after PDAC resection.^32^ Histogram, GLCM, and GWT features are textural features of collagen fibers identified in multiple studies, showing potential for clinical use in disease diagnosis.^30^, ^33^, ^34^ Quantifying collagen fibers can reveal microscopic features related to tumor metastasis that are not visible in standard medical imaging. Using ma‐TACS and mi‐TACS scores, we developed and validated a nomogram for predicting LN metastasis in PDAC patients. This nomogram, validated on an independent cohort, demonstrated reproducibility and reliability.

LN metastasis is a poor prognostic factor for PDAC. Compelling evidence highlights LN status as crucial for determining treatment options and predicting prognosis in pancreatic cancer patients. To improve long‐term survival, pancreatectomy is the most effective treatment, but debate continues over whether it should include standard or extended lymphadenectomy. However, more extensive pancreatectomies may lead to higher complication rates. For high‐risk patients, lymphadenectomy should be strongly considered during surgery. Therefore, predicting LN metastasis preoperatively or intraoperatively in PDAC may help avoid overtreatment and inform surgical decision‐making. CT, MRI, and PET are widely used for screening and diagnosing pancreatic cancer.^7^, ^8^ Surgical decisions largely rely on these imaging diagnostics, despite their performance not being fully satisfactory. While macroscopic features offer prognostic information for PDAC patients, they are insufficient for assessing LN status.^35^ Radiomics studies using these images are becoming more common. For example, Sun et al. integrated MRI and diffusion‐weighted imaging (DWI) to predict LN metastasis in pancreatic neuroendocrine tumors.^36^ Sin et al. developed and validated a similar MRI‐based radiomics model for predicting LN metastasis in PDAC, combining portal venous phase (PVP) contrast‐enhanced T1‐weighted imaging (T1WI) with T2‐weighted imaging (T2WI).^37^ Bian et al. developed an AI model with CT imaging to automatically predict LN metastasis in PDAC patients.^38^ These findings can guide treatment decisions to some extent. However, due to limited resolution and other objective factors, these imaging methods cannot evaluate histopathological features at the cellular and molecular levels as effectively as H&E staining, impacting the prediction accuracy and stability of the model.

Multiphoton microscopy achieves a resolution comparable to H&E staining, while SHG imaging provides significantly higher specificity to collagen fibers. According to Paget's seeds and soil hypothesis, the tumor microenvironment, or the soil surrounding the tumor seeds, is crucial for cancer development.^39^ Collagen fiber is a well‐known important component of the tumor microenvironment. Alterations in collagen structure and morphology play a significant role in solid tumors like breast and pancreatic cancer, increasing stiffness, invasion and metastasis.^27^, ^40^ Collagen network mechanical properties are influenced by factors such as fiber length, straightness, alignment, density and spatial orientation, possibly affecting stromal and tumor cell movement.^41^ The alignment of collagen fibers is a crucial factor in regulating tumor cell contact guidance. Different patterns of collagen alignment in the stroma of tumors are recognized and thought to facilitate tumor cell migration and invasion.^24^, ^27^, ^32^

In this study, we extracted both macroscopic and microscopic tumor‐associated collagen signatures (ma/mi‐TACS) from pancreatic ductal tumor tissues. Our findings indicate that both collagen signatures are significantly associated with LN metastasis in PDAC, with mi‐TACS showing a particularly strong correlation. This may indicate that microstructural changes in collagen fibers significantly influence tumor progression before macroscopic features form. Early stages of disease can result in microstructure changes that are not visually detectable. In the future, this method could be used for the differential diagnosis of early pancreatic tumors, improving early detection accuracy. To summarize, our observations indicate that ma‐TACS and mi‐TACS scores provide more precise predictions of LN metastasis compared to traditional clinical models. Additionally, these scores offer valuable insights into PDAC analysis, improving LN metastasis prediction based on collagen fibers. Despite these intriguing findings, this study has some limitations. Firstly, we developed a collagen‐based prediction model with a small sample size, based on a single‐center retrospective trial, limiting its application. Additional external validation with larger datasets is needed to confirm the model's robustness and accuracy. Secondly, the study focused solely on the presence or absence of LN metastasis in PDAC patients. However, the number of metastatic LNs is crucial, according to the latest cancer staging guidelines.

## 4 MATERIALS AND METHODS

### 4.1 Patients' characteristics

Our retrospective study received approval from the Institutional Review Board of The First Affiliated Hospital of Fujian Medical University, with informed consent waived. We collected 246 formalin‐fixed paraffin‐embedded (FFPE) PDAC tissue samples from patients aged 28 to 75 years. After excluding 96 samples based on the exclusion criteria, 150 samples passed quality control and were included in the final analysis. These samples were then randomly divided into two groups: 92 for the training cohort and 58 for the validation cohort. The inclusion criteria were: histologically confirmed PDAC patients; those with no prior radiotherapy or chemotherapy; and patients who had undergone pancreaticoduodenectomy with available pathological evidence of LN status. The exclusion criteria included: patients missing relevant clinicopathological characteristics; those with concurrent malignancies; damaged samples; or sections without tumor tissue. The detailed patient selection process is illustrated in Figure S7. Clinical characteristics obtained included gender, age at surgery, tumor size, differentiation grade, lymphovascular invasion, perineural invasion, and tumor location.

### 4.2 Sample preparation, multiphoton image acquisition

In this study, we utilized formalin‐fixed paraffin‐embedded (FFPE) tissues to gather a large number of samples along with pertinent clinical data. From the FFPE tissue samples, two consecutive 5‐μm thick sections were cut: one for MPM imaging and the other for H&E staining. The multiphoton microscopy system used in this study has been detailed in earlier publications. Briefly, we employed an upright microscope (LSM 880, Zeiss, Germany) coupled with a mode‐locked femtosecond Ti: sapphire laser (Chameleon Ultra, Coherent) to capture high‐resolution images. Optical imaging was performed using 810 nm linearly polarized light and a Plan‐Apochromat ×20 objective (NA = 0.8, Zeiss, Germany) for image acquisition. The imaging system provided submicron resolution, with lateral and axial resolutions around 0.3 and 0.8 μm, respectively. The backscattered signals were captured using two simultaneous channels: one for detecting second harmonic generation (SHG) signals, collected in the 395–415 nm range (green), and the other for two‐photon excitation fluorescence (TPEF) signals, collected in the 428–695 nm range (red). An average laser power of ~50 mW was applied to the sample, under which no observable photodamage occurred. To create a large‐scale image, a fine focusing stage was used to move the samples, stitching together a series of x–y scan images. Each x–y scan image had 512 × 512 pixels and a data depth of 12 bits.

### 4.3 Selection of regions of interest, collagen feature extraction, signature score construction

There were several non‐overlapping regions of interest (ROIs) numbered throughout the entire H&E image (Figure 1) across the invasive margin and adjacent tumor area by two pathologists who were blind to the patients' pathological outcomes. Label‐free MPM was used to simultaneously collect SHG and TPEF images for all annotated ROIs on another unstained section.

In our previous study, we provided a comprehensive protocol for quantifying macroscopic tumor‐associated collagen signatures (ma‐TACS).^27^ TACS1‐8 represents the macroscopic patterns of collagen fiber distribution both within the tumor center and at the tumor‐stromal interface. A detailed description of TACS1‐8 is provided in Table S5. Using the quantified TACS and LN status from the training cohort, ridge regression with cross‐validation was employed to determine the coefficients for each TACS. The coefficients for all TACS were incorporated into a formula to calculate a ma‐TACS score for each patient in both cohorts.

Following this, we intercepted regions of interest, each with a 512 × 512 pixels field of view, from each large‐scale MPM image to extract mi‐TACS. Four types of mi‐TACS were extracted using Matlab 2022a, including 8 morphologic features, 6 histogram‐based features, 80 gray‐level co‐occurrence matrix (GLCM) features, and 48 Gabor wavelet transform (GWT) features. Using a Gaussian mixture model segmentation algorithm, we initially segmented collagen fibers from the background in SHG images.^42^ Then, we applied a fiber network extraction algorithm to the binary mask image of collagen fibers to trace each fiber and identify cross‐link points, which are junctions connecting multiple fibers. The collagen area in an image is calculated by dividing the collagen pixels by the total pixels. The identified cross‐link points were utilized to calculate fiber density, length, width, straightness, crosslink density, crosslink space. Additionally, the collagen alignment orientation index was quantified using Fourier transform analysis.^43^ SHG pixel intensity histogram features described intensity distributions, such as mean, variance, skewness, kurtosis, energy, and entropy. The GLCM characterizes include four types of statistical features: contrast, correlation, energy, homogeneity. For the GWT features, we employed Gabor filters to determine the mean and variance of the convolution magnitude on the SHG images. The 142 mi‐TACS are not all related to LN metastasis, and some unrelated features may potentially degrade the predictive performance of the model. The feature selection of high‐dimensional data helps eliminate redundant features and retain the most pertinent ones for constructing a more effective predictive model. The least absolute shrinkage and selection operator (LASSO) is a widely used variable selection method that retains valuable variables while preventing overfitting. In order to build a reliable mi‐TACS score, we applied the LASSO regression to identify the most relevant and robust features out of 142 features. A new collagen signature was formed by linearly combining the selected features, with each feature weighted according to its coefficient.

### 4.4 Statistical analysis

Univariate and multivariate logistic regression analyses were conducted to assess conventional clinical risk factors, ma‐TACS score, and mi‐TACS score, aiming to investigate their relationships with LN metastasis (LN0, negative; LN1, positive). We evaluated the discriminatory performance of the developed models by utilizing ROC curves and calculating the AUC values. Additionally, the performance of the models was validated in the validation cohort. The nomogram was constructed using the training cohort and subsequently validated in the validation cohort, offering clinicians an intuitive and quantitative tool for rapid prediction of patient outcomes. The accuracy of the nomogram model was assessed using a calibration chart, which graphically depicts the correlation between observed probabilities and predicted probabilities. The alignment of the calibration curve with the diagonal line in the graph indicates the model's predictive accuracy. Patients were divided into LN0 and LN1 groups based on a threshold determined by the maximal Youden Index from the training cohort. We then applied the optimal sensitivity, specificity, and cutoff value derived from this index to the validation cohort. The χ^2^ test compared clinical categorical variables, whereas the Mann–Whitney *U* test analyzed differences in collagen signatures. DCA was used to evaluate clinical values by calculating the net benefits of different threshold probabilities for clinical, ma‐TACS, mi‐TACS, and nomogram models. The statistical analysis was performed with R 3.5.2 and IBM SPSS Statistics 25.

## 5 CONCLUSION

In conclusion, our preliminary results indicate that ma‐TACS and mi‐TACS scores from MPM images can accurately differentiate LN status in PDAC patients. Our developed and validated nomogram model may be valuable for prognosis and treatment management in PDAC patients.

## AUTHOR CONTRIBUTIONS

Gangqin Xi and Shuangmu Zhuo conceived the project and wrote or revised the manuscript. Shuangmu Zhuo, Lianhuang Li, Guozhong Liu and Jianxin Chen supervised the research. Gangqin Xi, Xiwen Chen, Yuhang Huang, Jiajia He, and Xiaolu Li performed multiphoton imaging. Linying Chen, Junyang Luo, Guozhong Liu, were responsible for sample collection, preparation and diagnosis. Gangqin Xi, Xiwen Chen, and Lianhuang Li conducted data analysis.

## CONFLICT OF INTEREST STATEMENT

The authors declare that they have no conflict of interest.

## Supporting information


**Table S1.** Characteristics of patients with pancreatic ductal adenocarcinoma in the training and validation cohorts.
**Table S2.** Univariate and multivariate logistic regression analyses of the association of variables with lymph node metastasis.
**Table S3.** The performance comparison of different models for predicting lymph node metastasis.
**Table S4.** Prediction of clinicopathologically classified patients by the ma‐TACS + mi‐TACS score.
**Table S5.** Detailed description about TACS1‐8.
**Figure S1.** Mi‐TACS selection using LASSO cox regression analysis. (A) Plot showing the relationship between the binomial deviance and log (λ). Dotted vertical lines are drawn at the optimal values by using the minimum criteria and the one standard error of the minimum criteria (the 1‐SE criteria). (B) Plot showing the relationship between the LASSO coefficient and log (λ). A dotted vertical line is the optimal lambda results with six nonzero coefficients.
**Figure S2.** (A) Box‐plots of Ma‐TACS score distribution for LN0 and LN1. (B) Box‐plots of Mi‐TACS score distribution for LN0 and LN1.
**Figure S3.** The ROC curves of the clinical model (A, B) and ma‐TACS score (C, D) in the training and validation cohorts.
**Figure S4.** The ROC curves of the mi‐TACS score model (E, F) and ma/mi‐TACS score (G, H) in the training and validation cohorts.
**Figure S5.** The ROC curves of the full model (clinical + ma/mi‐TACS score) (I, J) in the training and validation cohorts.
**Figure S6.** Decision curve analysis (DCA) for each model in both the training (A) and validation (B) cohorts. The y‐axis indicates the net benefit. The green, orange, red, and black line denotes the ma/mi‐TACS, mi‐TACS, ma‐TACS, and the clinical models, respectively. Gray denotes the “treat all” scheme and the blue denotes the “treat none” scheme.
**Figure S7.** Flow diagram showing inclusion and exclusion criteria for the study.

## Data Availability

The data that support the findings of this study are available from the corresponding author upon reasonable request.
