# Supplementary material for: Association of tumor associated collagen signature with lymph node metastasis in pancreatic ductal adenocarcinoma
Source: Bioeng Transl Med. 2025 Oct 30;11(3):e70087. doi: 10.1002/btm2.70087 (PMC13247407; doi:10.1002/btm2.70087)
Supplement: Supplementary file 1 — Table S1. Characteristics of patients with pancreatic ductal adenocarcinoma in the training and validation cohorts. Table S2. Univariate and multivariate logistic regression analyses of the association of variables with lymph node metastasis. Table S3. The performance comparison of different models for predicting lymph node metastasis. Table S4. Prediction of clinicopathologically classified patients by the ma‐TACS + mi‐TACS score. Table S5. Detailed description about TACS1‐8. Figure S1. Mi‐TACS selection using LASSO cox regression analysis. (A) Plot showing the relationship between the binomial deviance and log (λ). Dotted vertical lines are drawn at the optimal values by using the minimum criteria and the one standard error of the minimum criteria (the 1‐SE criteria). (B) Plot showing the relationship between the LASSO coefficient and log (λ). A dotted vertical line is the optimal lambda results with six nonzero coefficients. Figure S2. (A) Box‐plots of Ma‐TACS score distribution for LN0 and LN1. (B) Box‐plots of Mi‐TACS score distribution for LN0 and LN1. Figure S3. The ROC curves of the clinical model (A, B) and ma‐TACS score (C, D) in the training and validation cohorts. Figure S4. The ROC curves of the mi‐TACS score model (E, F) and ma/mi‐TACS score (G, H) in the training and validation cohorts. Figure S5. The ROC curves of the full model (clinical + ma/mi‐TACS score) (I, J) in the training and validation cohorts. Figure S6. Decision curve analysis (DCA) for each model in both the training (A) and validation (B) cohorts. The y‐axis indicates the net benefit. The green, orange, red, and black line denotes the ma/mi‐TACS, mi‐TACS, ma‐TACS, and the clinical models, respectively. Gray denotes the “treat all” scheme and the blue denotes the “treat none” scheme. Figure S7. Flow diagram showing inclusion and exclusion criteria for the study. [file BTM2-11-e70087-s001.docx]

**Supporting Information**

**Association of** **tumor associated collagen signature with lymph node metastasis in pancreatic ductal adenocarcinoma**

**Gangqin Xi^1^, Linying Chen^2^, Xiwen Chen^3^, Yuhang Huang^1^, Junyang Luo^4^, Jiajia He^1^, Xiaolu Li^1^, Jianxin Chen^3^, Guozhong Liu^5,^ *, Lianhuang Li^3,^ * and** **Shuangmu Zhuo^1,^ ***

^1^School of Science, Jimei University, Xiamen, China

^2^Department of Pathology, the First Affiliated Hospital of Fujian Medical University, Fuzhou, China.

^3^Key Laboratory of OptoElectronic Science and Technology for Medicine of Ministry of Education, Fujian Provincial Key Laboratory of Photonics Technology, Fujian Normal University, Fuzhou, China

^4^Department of Clinical Medicine, Xiamen Medical College, Xiamen, China

^5^Department of Hepatopancreatobiliary Surgery, the First Affiliated Hospital of Fujian Medical University, Fuzhou, China.

These authors contributed equally: Gangqin Xi, Linying Chen.

*Correspondence: Guozhong Liu (lgzpt090413@163.com); Lianhuang Li (lhli@fjnu.edu.cn); Shuangmu Zhuo (shuangmuzhuo@gmail.com).

**Table S1** Characteristics of patients with Pancreatic Ductal Adenocarcinoma in the training and validation cohorts.

| **Characteristic** | **Training cohort (92)** | **Validation cohort (58)** | ***P-*value** | **Total cohort (150)** |
| --- | --- | --- | --- | --- |
| **Age (y)** |  |  | 0.180 |  |
| ≤60 | 50 (54.3%) | 25 (43.1%) |  | 75 (50.0%) |
| >60 | 42 (45.7%) | 33 (56.9%) |  | 75 (50.0%) |
| **Gender** |  |  | 0.379 |  |
| Male | 30 (32.6%) | 23 (39.7%) |  | 53 (35.3%) |
| Female | 62 (67.4%) | 35 (60.3%) |  | 97 (64.7%) |
| **T category** |  |  | 0.735 |  |
| T1 | 13 (14.1%) | 6 (10.3%) |  | 19 (12.7%) |
| T2 | 49 (53.3%) | 34 (58.6%) |  | 83 (55.3%) |
| T3 | 30 (32.6%) | 18 (31.1%) |  | 48 (32.0%) |
| **Differentiation grade** |  |  | 0.504 |  |
| G1 | 14 (15.2%) | 13 (22.4%) |  | 27 (18.0%) |
| G2 | 39 (42.4%) | 21 (36.2%) |  | 60 (40.0%) |
| G3 | 39 (42.4%) | 24 (41.4%) |  | 63 (42.0%) |
| **Lymphovascular invasion** |  |  | 0.432 |  |
| Negative | 73 (79.3%) | 49 (84.5%) |  | 122 (81.3%) |
| Positive | 19 (20.7%) | 9 (15.5%) |  | 28 (18.7%) |
| **Perineural invasion** |  |  | 0.476 |  |
| Negative | 22 (23.9%) | 11 (19.0%) |  | 33 (22.0%) |
| Positive | 70 (76.1%) | 47 (81.0%) |  | 117 (78.0%) |
| **Tumor location** |  |  | 0.705 |  |
| Head1 | 64 (69.6%) | 44 (75.9%) |  | 108 (72.0%) |
| Body/tail2 | 24 (26.1%) | 12 (20.7%) |  | 36 (24.0%) |
| Other3 | 4 (4.3%) | 2 (3.4%) |  | 6 (4.0%) |
| **LN status** |  |  | 0.455 |  |
| Negative | 47 (51.1%) | 26 (44.8%) |  | 73 (48.7%) |
| Positive | 45 (48.9%) | 32 (55.2%) |  | 77 (51.3%) |

Abbreviations: LN, lymph node.

**Table S2.** Univariate and multivariate logistic regression analyses of the association of variables with lymph node metastasis.

| **Variable** | **Univariate analysis** | | | |  | **Multivariate analysis** | | | |
| --- | --- | --- | --- | --- | --- | --- | --- | --- | --- |
|  | **OR** | **(95%CI)** | | ***P*-value** |  | **OR** | **(95%CI)** | | ***P*-value** |
| Characteristic |  |  |  |  |  |  |  |  |  |
| **Age (y), mean (SD)** | 0.997 | 0.969 | 1.026 | 0.827 |  | 1.017 | 0.974 | 1.061 | 0.453 |
| **Gender** |  |  |  |  |  |  |  |  |  |
| Male |  |  |  |  |  |  |  |  |  |
| Female | 0.721 | 0.367 | 1.413 | 0.340 |  | 0.418 | 0.155 | 1.132 | 0.086 |
| **T category** |  |  |  |  |  |  |  |  |  |
| T1 |  |  |  |  |  |  |  |  |  |
| T2 | 0.821 | 0.300 | 2.247 | 0.700 |  | 0.870 | 0.206 | 3.676 | 0.849 |
| T3 | 0.615 | 0.210 | 1.800 | 0.375 |  | 0.820 | 0.167 | 4.025 | 0.807 |
| **Differentiation grade** |  |  |  |  |  |  |  |  |  |
| G1 |  |  |  |  |  |  |  |  |  |
| G2 | 1.336 | 0.537 | 3.328 | 0.534 |  | 0.589 | 0.142 | 2.442 | 0.466 |
| G3 | 1.466 | 0.592 | 3.628 | 0.409 |  | 0.903 | 0.226 | 3.614 | 0.886 |
| **Lymphovascular space invasion** |  |  |  |  |  |  |  |  |  |
| Negative |  |  |  |  |  |  |  |  |  |
| Positive | 1.333 | 0.582 | 3.053 | 0.496 |  | 0.814 | 0.247 | 2.679 | 0.734 |
| **Perineural invasion** |  |  |  |  |  |  |  |  |  |
| Negative |  |  |  |  |  |  |  |  |  |
| Positive | 1.157 | 0.534 | 2.507 | 0.711 |  | 0.854 | 0.279 | 2.608 | 0.781 |
| **Tumor location** |  |  |  |  |  |  |  |  |  |
| Head1 |  |  |  |  |  |  |  |  |  |
| Body/tail2 | 0.509 | 0.236 | 1.100 | 0.086 |  | 0.333 | 0.099 | 1.114 | 0.074 |
| Other3 | 0.800 | 0.154 | 4.144 | 0.790 |  | 0.350 | 0.037 | 3.303 | 0.359 |
| **Ma-TACS score** | 2.304 | 1.412 | 3.761 | 0.001 |  | 2.934 | 1.409 | 6.108 | 0.004 |
| **Mi-TACS score** | 3.325 | 2.296 | 4.814 | <0.001 |  | 3.861 | 2.488 | 5.993 | <0.001 |

**Table S3** The performance comparison of different models for predicting lymph node metastasis.

| **Model** | **Training cohort** | | | **Validation cohort** | | | |  |
| --- | --- | --- | --- | --- | --- | --- | --- | --- |
|  | **AUC (95%)** | **SEN (95%)** | **SPE (95%)** | | **AUC (95%)** | **SEN (95%)** | **SPE (95%)** |  |
| **CLI** | 64.2 (53.5 -73.9) | 80.0 (65.4 -90.4) | 53.2 (38.1 -67.9) | | 57.2 (43.5 -70.1) | 53.1 (34.7 -70.9) | 46.2(26.6 -66.6) |  |
|  |  |  |  |  |  |  |  |  |
| **Ma-TACS** | 68.7 (58.7 -77.9) | 66.7 (51.0 -80.0) | 66.0 (50.7 -79.1) | | 63.6 (49.9 -75.8) | 50.0 (31.9 -68.1) | 73.1 (52.2 -88.4) |  |
|  |  |  |  |  |  |  |  |  |
| **Mi-TACS** | 88.4 (80.0 -94.1) | 93.3 (81.7 -98.6) | 74.5 (59.7 -86.1) | | 83.1 (70.9 -91.6) | 87.5 (71.0 -96.5) | 76.9 (56.4 -91.0) |  |
|  |  |  |  |  |  |  |  |  |
| **Ma/mi-TACS** | 91.8 (84.2 -96.5) | 91.1 (78.8 -97.5) | 76.6 (62.0 -87.7) | | 83.1 (70.9 -91.6) | 81.3 (63.6-92.8) | 76.9 (56.4 -91.0) |  |
|  |  |  |  |  |  |  |  |  |
| **Full model** | 93.1 (85.9 -97.3) | 91.1 (78.8 -97.5) | 83.0 (69.2 -92.4) | | 81.9 (69.5-90.7) | 65.6 (46.8 -81.4) | 80.8 (60.6 -93.4) |  |

Note. Ma/mi-TACS indicates the combination of the ma-TACS score and the mi-TACS score. Full model indicates the combination of the clinical model, ma-TACS score, and mi-TACS score. Abbreviations: AUC, area under the receiver operating characteristic curve; SEN, sensitivity; SPE, specificity.

**Table S4** Prediction of clinicopathologically classified patients by the ma-TACS + mi-TACS score.

| **Subgroups** | **Predict LN0 (73)** | **Predict LN1 (77)** | **OR** | ***P-*value** | **AUC** | **SEN** | **SPE** | **PPV** | **NPV** | **ACC** |
| --- | --- | --- | --- | --- | --- | --- | --- | --- | --- | --- |
| **Age (y)** |  |  |  |  |  |  |  |  |  |  |
| ≤60 | 36(49.3) | 39(50.6) | 1.982 | <0.001 | 0.895 | 0.948 | 0.750 | 0.804 | 0.931 | 0.853 |
| >60 | 37(50.7) | 38(49.4) | 1.987 | <0.001 | 0.866 | 0.789 | 0.784 | 0.789 | 0.784 | 0.787 |
| **Gender** |  |  |  |  |  |  |  |  |  |  |
| Male | 23(31.5) | 30(39.0) | 2.208 | <0.001 | 0.880 | 0.767 | 0.870 | 0.885 | 0.741 | 0.811 |
| Female | 50(68.5) | 47(61.0) | 2.015 | <0.001 | 0.889 | 0.936 | 0.72 | 0.759 | 0.923 | 0.825 |
| **T category** |  |  |  |  |  |  |  |  |  |  |
| T1 | 8(11.0) | 11(14.3) | 2.624 | 0.022 | 0.864 | 1.000 | 0.75 | 0.846 | 1.000 | 0.895 |
| T2 | 39(53.4) | 44(57.1) | 1.994 | <0.001 | 0.885 | 0.818 | 0.821 | 0.837 | 0.800 | 0.819 |
| T3 | 26(35.6) | 22(28.6) | 1.900 | 0.001 | 0.888 | 0.909 | 0.692 | 0.714 | 0.900 | 0.792 |
| **Differentiation grade** |  |  |  |  |  |  |  |  |  |  |
| G1 | 15(20.6) | 12(15.6) | 1.622 | 0.013 | 0.839 | 0.667 | 0.800 | 0.727 | 0.750 | 0.741 |
| G2 | 29(39.7) | 31(40.3) | 1.947 | <0.001 | 0.892 | 0.935 | 0.759 | 0.806 | 0.917 | 0.850 |
| G3 | 29(39.7) | 34(44.2) | 2.699 | <0.001 | 0.900 | 0.882 | 0.759 | 0.811 | 0.846 | 0.825 |
| **Lymphovascular invasion** |  |  |  |  |  |  |  |  |  |  |
| Negative | 61(83.6) | 61(79.2) | 2.237 | <0.001 | 0.898 | 0.852 | 0.803 | 0.812 | 0.845 | 0.828 |
| Positive | 12(16.4) | 16(20.8) | 1.394 | 0.060 | 0.776 | 0.938 | 0.583 | 0.750 | 0.875 | 0.786 |
| **Perineural invasion** |  |  |  |  |  |  |  |  |  |  |
| Negative | 17(23.3) | 16(20.8) | 2.828 | 0.003 | 0.901 | 0.938 | 0.824 | 0.833 | 0.933 | 0.879 |
| Positive | 56(76.7) | 61(79.2) | 1.859 | <0.001 | 0.870 | 0.852 | 0.750 | 0.788 | 0.824 | 0.803 |
| **Tumor location** |  |  |  |  |  |  |  |  |  |  |
| Head1 | 48(65.8) | 60(77.9) | 1.926 | <0.001 | 0.883 | 0.850 | 0.812 | 0.850 | 0.812 | 0.833 |
| Body/tail2 | 22(30.1) | 14(18.2) | 2.659 | 0.002 | 0.906 | 0.929 | 0.682 | 0.650 | 0.938 | 0.778 |
| Other3 | 3(4.1) | 3(3.9) | 18.076 | 0.529 | 0.889 | 1.000 | 0.667 | 0.750 | 1.000 | 0.833 |

**Table S5** Detailed description about TACS1-8

| **TACS1-8** | **Feature description** |
| --- | --- |
| TACS1 | collagen fibers curved and elastically wrapped the tumor nest during the tumorigenesis stage. |
| TACS2 | collagen fibers stretched due to the growth of tumors, wrapped around the tumor but aligned parallel to the boundary. |
| TACS3 | collagen fibers are perpendicular to the tumor nest boundary, radiating outside the duct, and tumor cells may migrate along the collagen fibers. |
| TACS4 | At the border of the tumor, the tumors invade in an expansive manner, and collagen fibers distributed in a reticular pattern. |
| TACS5 | At the tumor boundary, the distribution of collagen fibers has obvious directionality. The direction of collagen fibers is parallel to the tumor invasion direction, which provides a way for tumor invasion. |
| TACS6 | At the tumor boundary, tumors invade in a disordered and chaotic manner, and the distribution of collagen fibers is consistent with the direction of invasion of tumors. |
| TACS7 | collagen fibers are densely distributed at the tumor invasion front largely free of tumors cells. |
| TACS8 | collagen fibers are sparsely distributed at the tumor invasion front largely free of tumors cells. |

**Ma-TACS score and mi-TACS score calculation formulas**

Using the ridge regression analysis, we obtain a TACS-score for each patient based on the

combined TACS1-8.

Ma-TACS score =-1.4663464+ (0.9970001 * TACS1) + (10.1951931 * TACS2) + (– 1.9484164 * TACS3) + (0.4167665 * TACS4) + (1.5025524 * TACS5) + (2.2413038 * TACS6) + (0.9657341 * TACS7) + (0.2288334 * TACS8)

Using the LASSO logistic regression analysis, we obtain a mi-TACS score for each patient based on the six selected microscopic features.

Mi-TACS score =-0.06864118 +(-0.09851105 * Length) +(0.64171781 * Straightness) +(0.54252351 * Orientation) +(0.78679901 * Kurtosis of histograms) +(-0.46889455 * GLCM correlation_135°_5 pixel) +(-0.06386891 * Gabor_ variance_120°_2 scale)

**Figure S1.** Mi-TACS selection using LASSO cox regression analysis. (A) Plot showing the relationship between the binomial deviance and log (λ). Dotted vertical lines are drawn at the optimal values by using the minimum criteria and the one standard error of the minimum criteria (the 1-SE criteria). (B) Plot showing the relationship between the LASSO coefficient and log (λ). A dotted vertical line is the optimal lambda results with 6 nonzero coefficients.

**
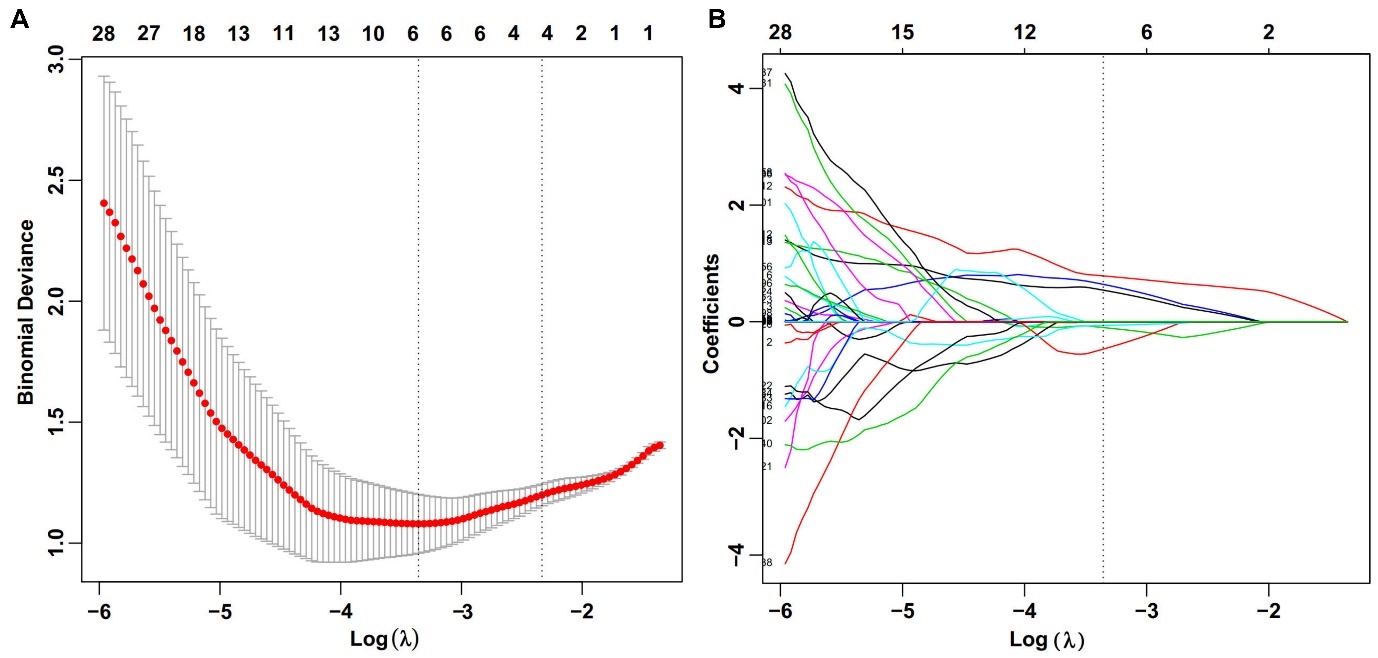
**

**Figure S2.** (A) Box-plots of Ma-TACS score distribution for LN0 and LN1. (B) Box-plots of Mi-TACS score distribution for LN0 and LN1.

**
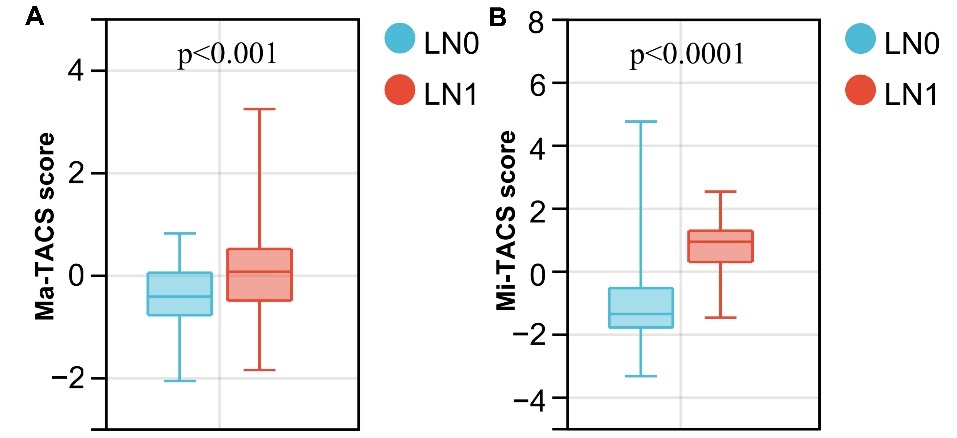
**

**Figure S3.** The ROC curves of the clinical model (A, B) and ma-TACS score (C, D) in the training and validation cohorts.

**
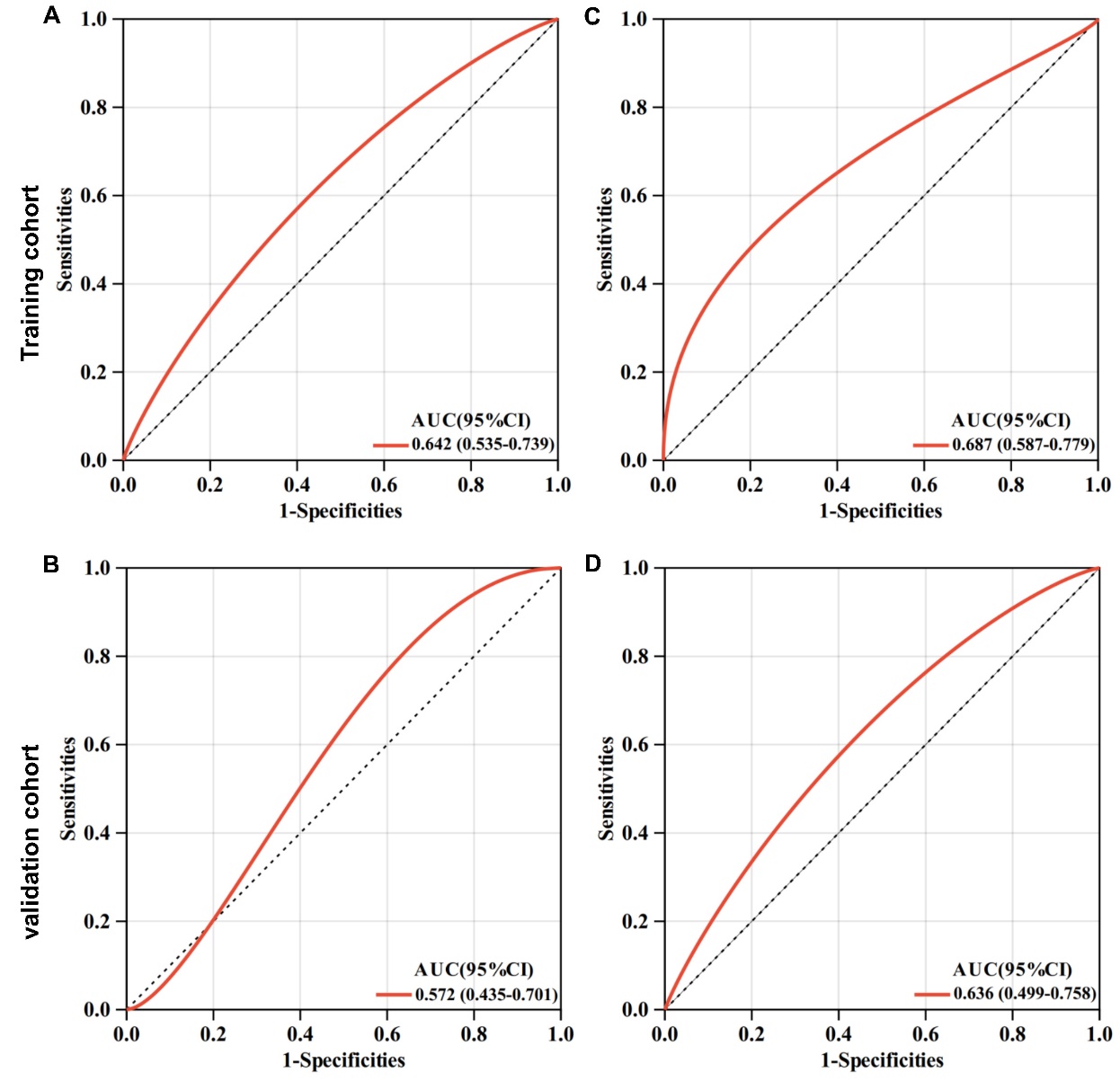
**

**Figure S4.** The ROC curves of the mi-TACS score model (E, F) and ma/mi-TACS score (G, H) in the training and validation cohorts.

**
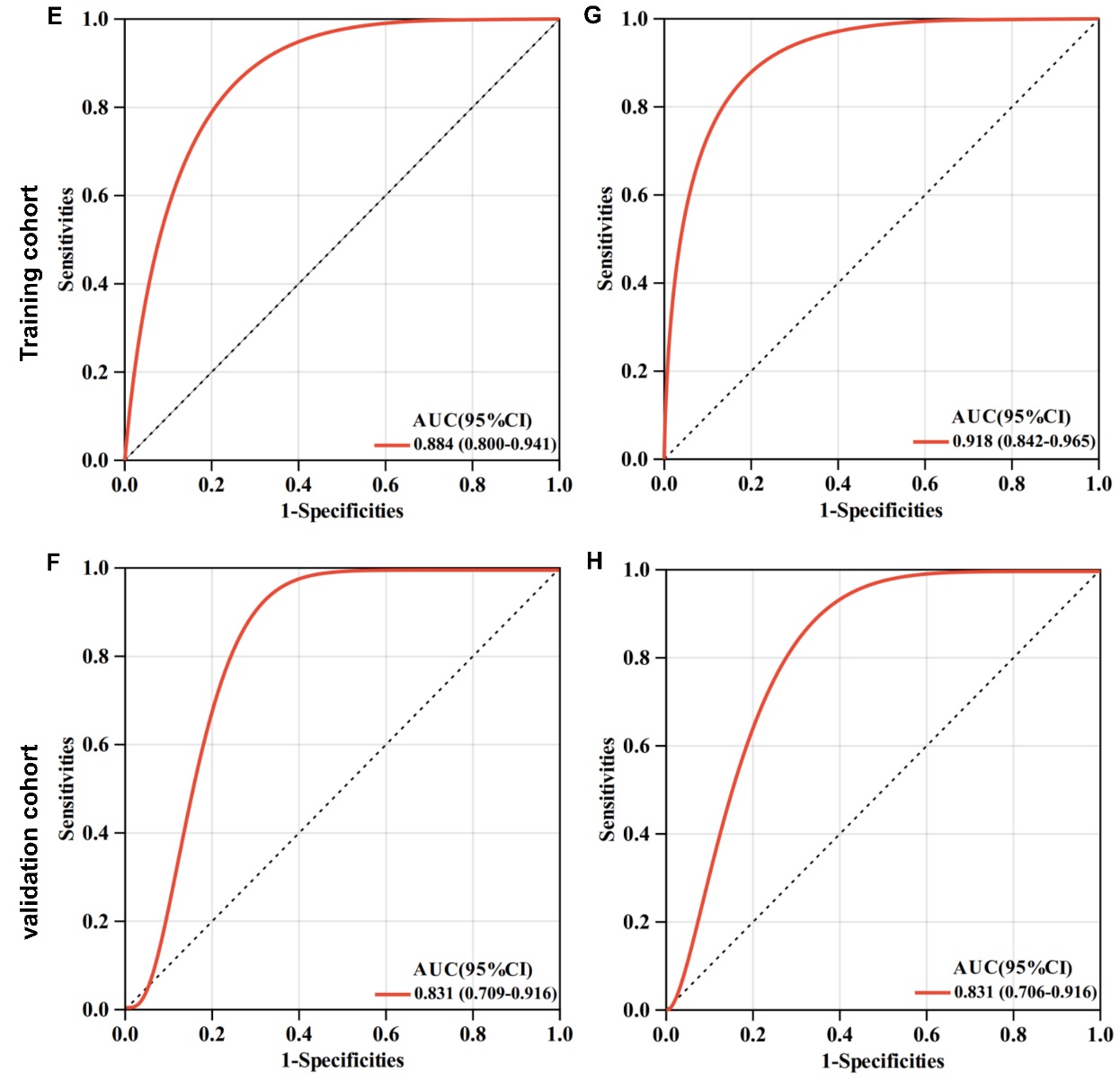
**

**Figure S5.** The ROC curves of the full model (clinical + ma/mi-TACS score) (I, J) in the training and validation cohorts.

**
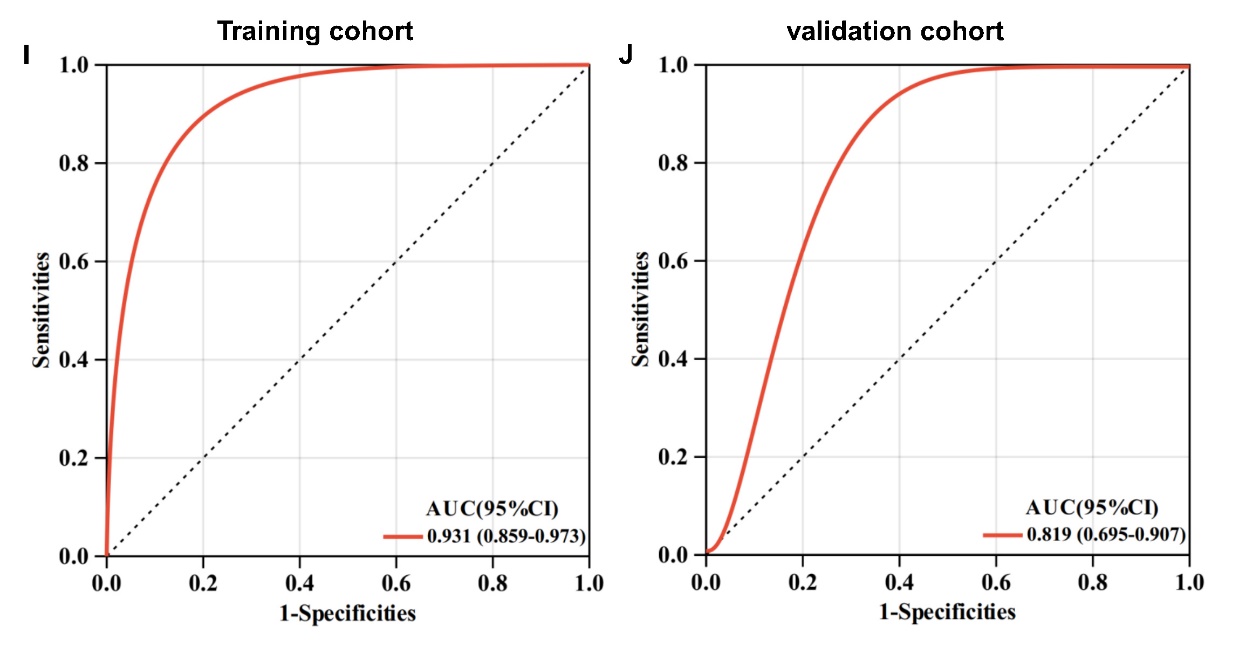
**

**Figure S6** Decision curve analysis (DCA) for each model in both the training (A) and validation (B) cohorts. The y-axis indicates the net benefit. The green, orange, red, and black line denotes the ma/mi-TACS, mi-TACS, ma-TACS, and the clinical models, respectively. Gray denotes the “treat all” scheme and the blue denotes the “treat none” scheme.

**
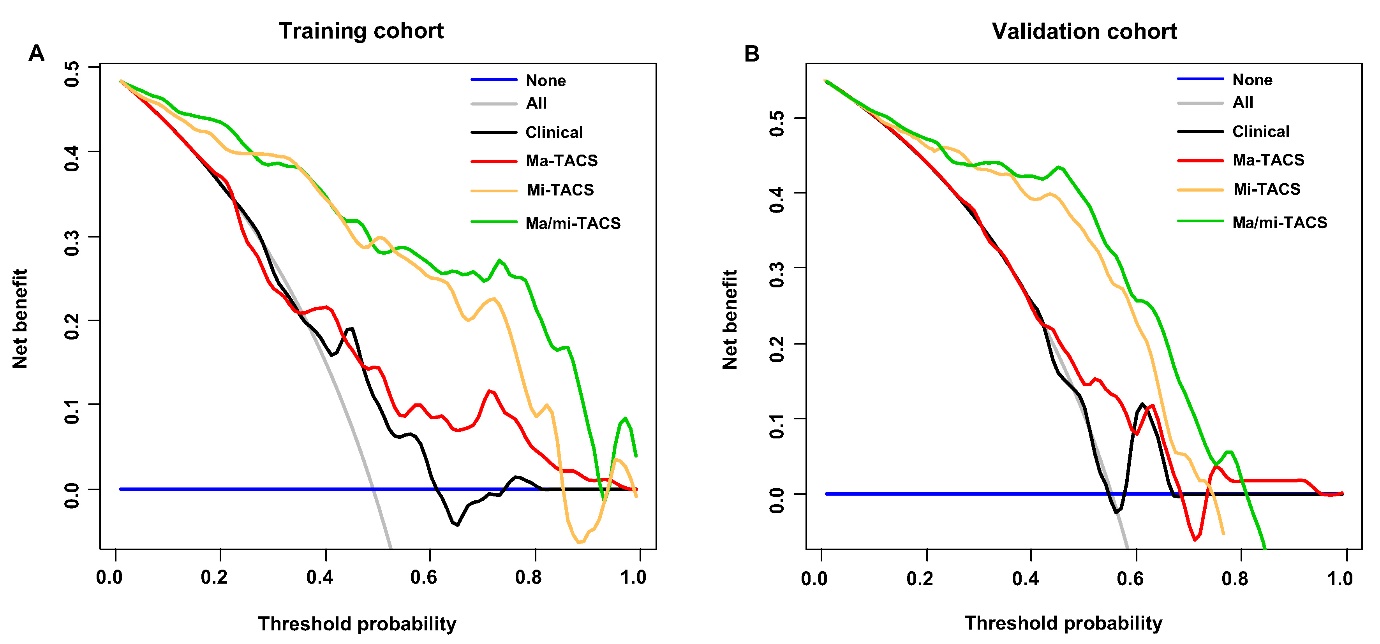
**

**Figure S7**. Flow diagram showing inclusion and exclusion criteria for the study.

**
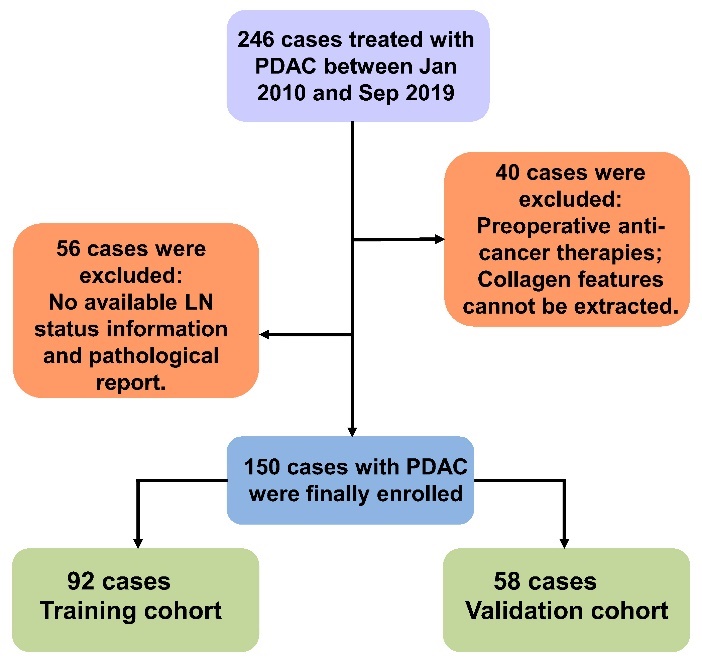
**
